# Synthesis of CdTe quantum dot-conjugated CC49 and their application for *in vitro* imaging of gastric adenocarcinoma cells

**DOI:** 10.1186/1556-276X-8-294

**Published:** 2013-06-22

**Authors:** Yun-Peng Zhang, Peng Sun, Xu-Rui Zhang, Wu-Li Yang, Cheng-Shuai Si

**Affiliations:** 1 Department of General Surgery, Huashan Hospital, Fudan University, Shanghai 200040, China; 2 State Key Laboratory of Molecular Engineering of Polymers and Department of Macromolecular Science, Fudan University, Shanghai 200433, China

**Keywords:** Stomach neoplasms, Quantum dots, Nanotechnology

## Abstract

The purpose of this experiment was to investigate the visible imaging of gastric adenocarcinoma cells *in vitro* by targeting tumor-associated glycoprotein 72 (TAG-72) with near-infrared quantum dots (QDs). QDs with an emission wavelength of about 550 to 780 nm were conjugated to CC49 monoclonal antibodies against TAG-72, resulting in a probe named as CC49-QDs. A gastric adenocarcinoma cell line (MGC80-3) expressing high levels of TAG-72 was cultured for fluorescence imaging, and a gastric epithelial cell line (GES-1) was used for the negative control group. Transmission electron microscopy indicated that the average diameter of CC49-QDs was 0.2 nm higher compared with that of the primary QDs. Also, fluorescence spectrum analysis indicated that the CC49-QDs did not have different optical properties compared to the primary QDs. Immunohistochemical examination and *in vitro* fluorescence imaging of the tumors showed that the CC49-QDs probe could bind TAG-72 expressed on MGC80-3 cells.

## Background

Gastric cancer has ranked as one of the most frequent tumors in the world with approximately 989,000 new cases and 738,000 deaths per year [1]. The most important part in surgery is to correctly define the boundary of the tumor and precisely determine the regions for surgical resection in order to improve survival rate and quality of life. However, visualized methods to detect the tumor cells during surgery are currently not available. Both D1 lymphadenectomy proposed by Western researchers and D2 lymphadenectomy proposed by Japanese researchers cannot achieve high specificity [2]. Clinical doctors could only estimate the tumor boundary for surgical resection by experience and the changes of the tumor tissue texture, which results in a high failure rate of complete removal of gastric cancer and greatly affects the survival rate of the patients. Therefore, development of methods for real-time identification of tumor cells and metastasized lymph nodes during surgery and establishment of tailored surgical resection for each individual are one of the key factors in improving the survival rate for gastric cancer.

Recently, quantum dots (QDs) were developed on the interdisciplinary advancement of nanotechnology, chemistry, and optics. The unique optical properties of QDs have shown promising prospects in the tumor tissue and metastasized lymph node clearance for cancer patients [3]. Compared with traditional organic dyes, inorganic semiconductor QDs exhibit more advantages on light absorption, bright fluorescence, narrow symmetric emission bands, high photostability, and size-tunable optical properties and are considered to be valuable fluorescent probes for tissue imaging. Particularly, people pay close attention to near-infrared (NIR) QDs for visible *in vivo* tissue imaging due to their reduced absorbance and scattering in biological tissues within the NIR region, as well as the strong penetration in human tissues. The unique optical properties and the ease of modification of QDs by some bioactive materials make these nanoparticles as highly promising fluorescent labels for *in vivo* biological applications [4,5]. Currently, fluorescent probes have been developed by conjugating QDs with target molecules (e.g., antibodies and peptides) and have been used for *in vivo* visualization of cancer cells [6], sentinel lymph node detection [7,8], and imaging of drug targeting studies [9]. More important, new synthetic techniques of QDs biologically functionalized QDs with excellent biological compatibility and water solubility, which pave the way for the application of tissue imaging *in vivo*[10].

A common limitation of the QDs’ use in tissue imaging *in vivo* was their potential toxicity. Some researchers claimed that the oxidation of Cd^2+^ on the QD surface and subsequent Cd^2+^ release may induce potential cytotoxicity [11]. However, many authoritative studies showed that there was no significant influence on cell viability, morphology, function, or development in the use of QDs [12,13]. Besides, no obvious toxicity evidence was obtained during *in vivo* imaging [7,14-16]. In our previous experiments, CdTe quantum dots were proved not having acute toxicity to rats when they were injected in the subserosa layer of the rats’ stomach [17].

Many studies have demonstrated that 75% of gastric adenocarcinomas highly express tumor-associated glycoprotein 72 (TAG-72) [18]. Specific targeting of TAG-72 by CC49 antibodies has been widely used for the treatment of gastric cancer [19-21]. Therefore, visual imaging by targeting TAG-72 has broad applicability for gastric cancer detection. The authors of this research attached CC49 monoclonal antibodies to QDs with a maximal emission wavelength of 710 nm to produce a probe designated as CC49-QDs and reported the use of CC49-QDs as fluorescent probes for imaging the human gastric adenocarcinoma cell line MGC80-3.

## Methods

### Main equipment, reagents, and cell lines

Cadmium chloride (CdCl_2_), 3-mercaptopropionic acid (MPA), and sodium borohydride (NaBH_4_) were purchased from Acros Organics (Geel, Belgium). Tellurium powder was purchased from Sigma-Aldrich (St. Louis, MO, USA). *N*-(3-Dimethylaminopropyl)-*N*-ethylcarbodiimide hydrochloride (EDC) and *N*-hydroxysuccinimide (NHS) were supplied by Shanghai Medpep Co., Ltd. (Shanghai, China). The gastric cancer cell line MGC80-3 was supplied by Shanghai Institutes for Biological Sciences, Chinese Academy of Sciences (Shanghai, China). The human gastric epithelial cell line GES-1 was purchased from Beijing Institute for Cancer Research (Beijing, China). CC49 monoclonal antibody and secondary antibody were purchased from Santa Cruz Biotechnology (Santa Cruz, CA, USA). The fluorescence images of cells were obtained with a Nikon microscope (NIKON 80i, Tokyo, Japan) which was equipped with a high-definition CCD camera, a 160-W Hg excitation lamp, and three filters (*λ*_ex_ 380 nm/*λ*_em_ 420 nm, *λ*_ex_ 490 nm/*λ*_em_ 520 nm, and *λ*_ex_ 560 nm/*λ*_em_ 590 nm). Agilent 1200 and gel permeation column was purchased from Agilent (Santa Clara, CA, USA).

### Synthesis of CdTe QDs

The synthesis of CdTe QDs has been described in detail elsewhere [22]. Briefly, 100 mg sodium borohydride was used to react with 127 mg (1 mmol) tellurium in 20 ml of distilled water under an ice water bath condition to prepare sodium hydrogen telluride (NaHTe). With continuous steady nitrogen flow and vigorous stirring, a clear and purple NaHTe solution was successfully produced. To ensure that the mole ratio of Cd^2+^ to MPA was 1:1.8, 366.6 mg (2 mmol) CdCl_2_ and 382.1 mg (3.6 mmol) MPA were dissolved in 100 ml of water followed by adjustment of pH to 9.0 in the ice water bath. Then, 1 ml of oxygen-free solution containing fresh NaHTe, cooled to 0°C, was added to 20 ml of the above CdCl_2_-MPA solution and vigorously stirred. Finally, a Teflon-lined stainless steel autoclave with a volume of 9 ml of precursor solution was placed in a drying oven at 185°C. The precipitation products were washed with ethanol three times and then put into a vacuum drying oven at 40°C. Primary QDs were thus obtained. The photoluminescence (PL) quantum yield (QY) of CdTe QDs was estimated by comparison with Rhodamine 6G in ethanol at room temperature, assuming its PL QY as 45% [22]. X-ray diffraction (XRD) patterns of CdTe QDs were taken on a Rigaku D/MAX-IIA diffractometer (Shibuya-ku, Japan) using Cu KαR radiation [21].

### Synthesis of CC49-QDs

Preparation of CC49-QDs antibody (Ab) probes was performed according to instructions of the QD Antibody Conjugation Kits [23]. Briefly, 13.5 μl of EDC and 13.5 μl of NHS were mixed with a 50-μl CdTe QD solution and shaken for 0.5 h at room temperature. Then, 594 μl of CC49 monoclonal antibodies was added, resulting in a CdTe to antibody ratio of 1:4. Another 2 h was needed for the reaction at room temperature followed by centrifugation. The centrifugation was done four times using a 100K ultra filter at 5,000 rpm for 15 min. Each time, liquids at the lower strata were discarded, and the supernatant products were diluted by 200 μl of phosphate-buffered saline (PBS) before subsequent centrifugation. The final product was diluted with PBS (pH 7.4) and stored in a refrigerator at 4°C.

### QD and CC49-QDs electron microscopy and spectrum analysis

The prepared primary QDs and CC49-QDs were separately diluted in deionized water, and several drops were dropped onto two pieces of carbon films supported by a copper mesh. When the water volatilized, they were put under the electron microscope adjusted to a 200-V stem mode for observation. Diluted QDs and CC49-QDs were put under a spectrofluorimeter with a 450-nm excitation wavelength and a 1-mm slit. The curves of the spectra were drawn by recording the intensities of each nanometer of emission light between 550 and 800 nm.

### Gel permeation high-performance liquid chromatography

The CC49 and CC49-QDs were monitored by high-performance liquid chromatography (HPLC) gel filtration. Samples were injected onto a ZORBAX GF-450 (9.5 × 250, 6-μm size, Agilent) exclusion column connected in a series with 67 mM phosphate and 100 mM KCl buffer (pH 6.8) as a mobile phase at a flow rate of 1 ml/min. The absorption was monitored at 280 nm [24,25].

### Immunohistochemical detection of TAG-72

One milliliter of MGC80-3 cells and GES-1 at a concentration of 2 × 10^4^ cells/ml were separately seeded into each well of a 24-well plate containing a glass cover slip. After 24 h of culture, the cells were fixed with 4% paraformaldehyde for 20 min. Streptavidin peroxidase (SP) immunohistochemical staining was performed according to instructions of the Sunhis-H kits. Briefly, the cover slips were incubated with 3% H_2_O_2_ deionized water for 10 min, and washed with PBS two times (each for 3 min). Consequently, the cover slips were incubated with protein blocking working liquid at room temperature for 5 min before the CC49 monoclonal antibody (1:100) was added. After incubation overnight at 4°C, the cover slips were washed with PBS three times (each for 3 min), and then biotin-labeled goat antimouse immunoglobulin G was added. After 10 min, PBS was also used to wash the cover slips for three times (each for 5 min). Then, the streptavidin conjugate of horseradish peroxidase was added for incubation for another 10 min. Finally, diaminobenzidine was added for color development. The negative control group was prepared by adding PBS instead of the primary antibody. Brown-yellow granules that appeared in the cells indicated a positive result [26].

### *In vitro* immunofluorescence

MGC80-3 cells and GES-1 at a concentration of 5 × 10^4^ cells/ml were seeded separately onto four 35-mm culture dishes with glass bottoms (1 ml in each dish). The four 35-mm culture dishes of MGC80-3 were marked A, B, C, and D, while those of the GES-1 group were marked E, F, G, and H. After 24 h of culture, the cells were washed with PBS twice. The experimental dishes B and F were added with 100 μl of CC49-QDs Ab probe (337.5 nmol). The negative control dishes A and E were added 100 μl of QDs (337.5 nmol) for the purpose of insteading of the CC49-QDs Ab probe. The cells in the four dishes described above were incubated for 1 h at 37°C and then washed with PBS three times. The competitive group dishes C and G were added to 200 μl of CC49 monoclonal antibody (1 μg/ml) for 2 h of blocking. Subsequently, the cells were washed with PBS twice, and then an equimolar amount of CC49-QDs Ab probe was added to the experimental dishes. To the positive control dishes D and H, 100 μl of CC49 monoclonal antibody (1 μg/ml) was added for 2 h of blocking. After washing three times (each for 3 min), fluorescent secondary antibody (goat against mouse IgG and conjugated to fluorescein isothiocyanate, 1:100) was added for another 30 min of incubation. 4′,6-Diamidino-2-phenylindole (DAPI) was used to label the cell nucleus before imaging with a fluorescence microscope.

**Figure 1:**
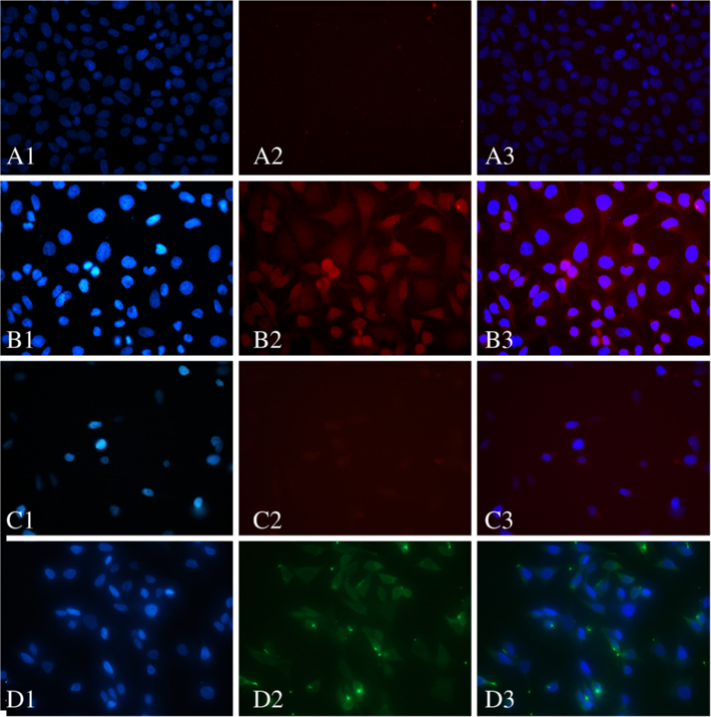
***In vitro *labeling of MGC80-3 cells with CC49-QDs Ab probe and primary QDs.** (A1/B1/C1/D1) The cell nucleus was stained with DAPI. (A2) MGC80-3 cells labeled with QDs. (B2) MGC80-3 cells labeled with CC49-QDs. (C2) MGC80-3 cells labeled with CC49-QDs after blocked with free CC49. (D2) MGC80-3 cells labeled with fluorescent secondary antibody. A3/B3/C3/D3 were merged with A1 and A2, B1 and B2, C1 and C2, D1 and D2, respectively.

**Figure 2:**
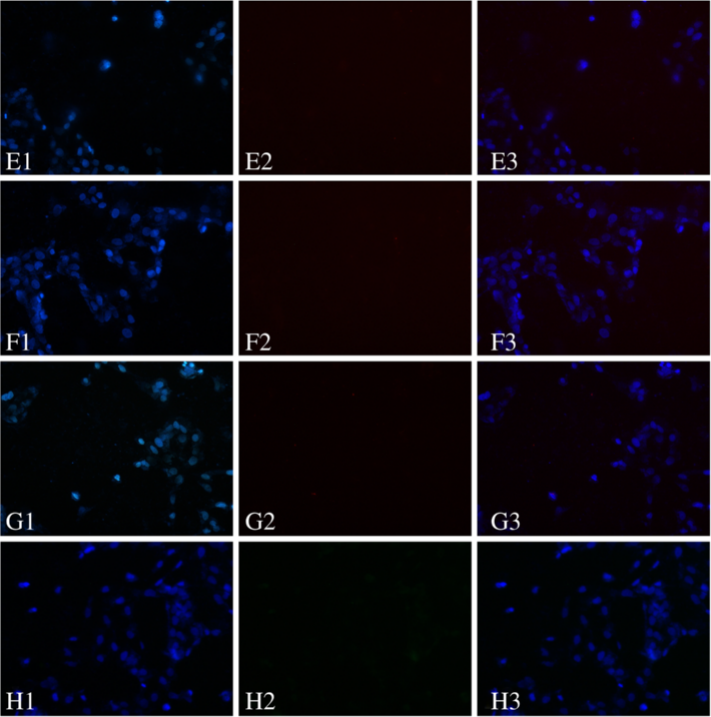
***In vitro *labeling of GES-1 cells with CC49-QDs Ab probe and primary QDs.** (E1/F1/G1/H1) The cell nucleus was stained with DAPI. (E2) GES-1 cells labeled with QDs. (F2) GES-1 cells labeled with CC49-QDs. (G2) GES-1 cells labeled with CC49-QDs after blocked with free CC49. (H2) GES-1 cells labeled with fluorescent secondary antibody. E3/F3/G3/H3 were merged with E1 and E2, F1 and F2, G1 and G2, H1 and H2, respectively.

In the fluorescence imaging of the cancer cells, the cell nucleus stained with DAPI (A1/B1/C1/D1 in Figure 1 and E1/F1/G1/H1 in Figure 2) was observed under the UV mode in which the excitation wavelength was 330 to 380 nm and the emission wavelength was 400 to 420 nm. MGC80-3 cells labeled with QDs (A2 in Figure 1) and CC49-QDS (B2 and C2 in Figure 1) were observed under the G-2A mode in which the excitation wavelength was 510 to 560 nm and the emission wavelength was 575 to 590 nm. GES-1 cells labeled with QDs (E2 in Figure 2) and CC49-QDS (F2 and G2 in Figure 2) were observed under the same mode. MGC80-3 cells (D2 in Figure 1) and GES-1 cells (H2 in Figure 2) labeled with fluorescent secondary antibody were imaged under the FITC mode in which the excitation wavelength was 465 to 490 nm and the emission wavelength was 505 to 520 nm. All the experiments were repeated three times.

## Results and discussion

### Synthesis of the QDs and CC49-QDs

**Figure 3:**
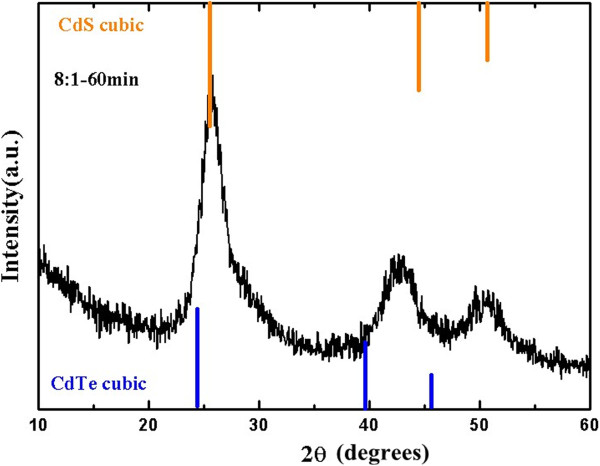
**Powder X-ray diffraction pattern of hydrothermally prepared CdTe QDs (*λ***_**cm **_**= 600 nm).** The line spectra show the cubic CdTe and CdS reflections with their relative intensities.

In this experiment, near-infrared water-soluble CdTe QDs (PL QY ≈ 41.6%) were synthesized by a hydrothermal route and were then characterized by XRD as shown in Figure 3. It is well known that the CdTe QDs belonged to a kind of core-shell CdTe/CdS structure. The XRD pattern showed that positions of CdTe QDs were intermediate between the values of cubic CdTe and CdS phases.

**Figure 4:**
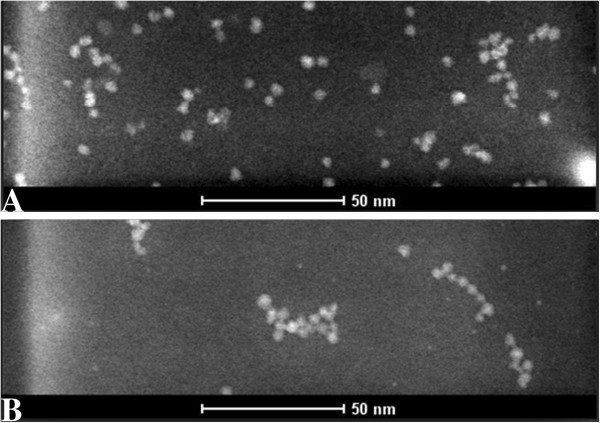
**Physical properties of near-infrared quantum dots.** (**A**) Transmission electron microscope image of QDs. (**B**) Transmission electron microscope image of CC49-QDs.

The electron microscope images (Figure 4) of QDs and CC49-QDs were obtained by transmission electron microscopy under the stem mode (200 V). The scale plate in the electron microscope system was used to measure all the QDs in a single visual field to get their average diameter and standard deviation. Then, it is the same for CC49-QDs. The images show that the average diameters of QDs and CC49-QDs were 3.5 ± 0.30 nm (Figure 4A) and 3.7 ± 0.31 nm (Figure 4B), respectively.

**Figure 5:**
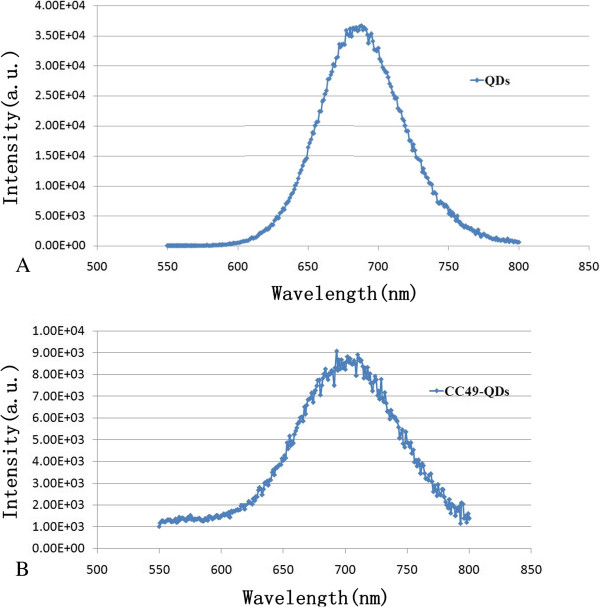
**Spectrum analysis.** (**A**) The primary CdTe QD spectrum analysis curve. (**B**) The CC49-QDs spectrum analysis curve.

With the ordinate denoting light intensity and the abscissa denoting wavelength, the spectrum curves for QDs and CC49-QDs were drawn. As shown in Figure 5, the emission wavelengths of primary QDs were between 580 and 800 nm, and the peak appeared around 680 nm (Figure 5A). The wavelengths of the CC49-QDs emission light were between 570 and 800 nm, and the peak appeared around 710 nm (Figure 5B). Also, the intensity of the CC49-QDs decreased about 75% as compared with that of the primary QDs, which may be caused by the loss of QDs during the centrifugation or the quench by CC49. Even so, the light is still much stronger than that of the organic dyes (Figure 1).

In the medical surgery of gastric cancer, determining the precise boundary of the tumors for individual surgical resection is the key to improve the survival rate of cancer patients. Traditional methods (e.g., computed tomography and magnetic resonance imaging) can provide good imaging in the detection of tumors but are not suitable for visible detection of tumor cells during surgery. Cancer cell imaging provides us a new way to develop the individual treatment for gastric cancer.

In tumor cell imaging, the ideal method should be sensitive, accurate, rapid, noninvasive, nonradioactive, and potentially useful in surgery. Previous cancer cell imaging usually involves a preoperative injection of a radioactive colloid tracer (e.g., ^99m^Tc sulfur colloid) followed by an intraoperative injection of a visible blue dye (e.g., isosulfan blue). However, these staining materials have deficits in imaging, such as poor tissue contrast and difficult detection in deep, dark anatomical regions. As to radioactive isotopes, the high radioactivity of the primary injection site can interfere with intraoperative *in vivo* detection of nearby lymph nodes [27,28]. For QDs, their unique optical properties have been mentioned earlier. More important, QDs can be easily modified and conjugated with other biological molecules. Conjugated QDs with good photochemical stability can easily penetrate tumor angiogenesis and access cancer cells. As a result, they possess unique advantages in the surgical treatment of individual cancer patients.

### Confirmation of conjugate formation

**Figure 6:**
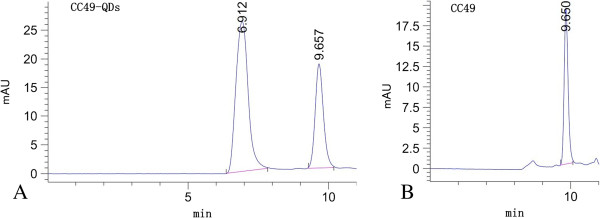
**HPLC elution curves for (A) CC49-QDs and (B) free CC49.** The retention times of CC49-QDs and free CC49 were about 6.91 and 9.65 min, respectively.

In the experiment, the formation of QD bioconjugates was confirmed by HPLC size-exclusion chromatography. As the species with higher molecular weights are eluted in shorter retention times, the HPLC peaks observed at retention time 9.65 min were attributed to free CC49 (Figure 6A,B). The molecular weight of the CC49 antibody is 150 kDa. After conjugation, being shifted to a higher molecular weight, the peaks can be observed at 6.91 min, as expected for the attachment of QDs to CC49 (Figure 6A).

### Immunohistochemical detection of TAG-72

**Figure 7:**
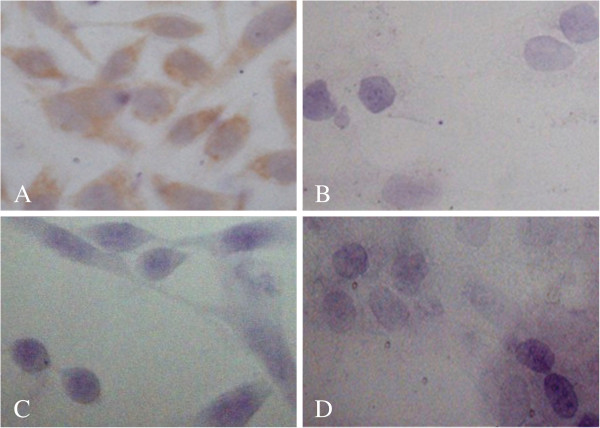
**Immunohistochemical examination of TAG-72 expression.** Experimental group: the SP immunohistochemical staining of MGC80-3 (**A**) and GES-1 (**B**). Control group (the primary antibody was replaced by PBS): the SP immunohistochemical staining of MGC80-3 (**C**) and GES-1 (**D**).

Immunohistochemical staining demonstrated that the CC49 monoclonal antibodies bound to TAG-72 of the MGC80-3 cells. As shown in Figure 7, positive staining (brown stain) was observed for the MGC80-3 cells of the CC49 antibody group (Figure 7A), as expected, indicating that TAG-72 is highly expressed in these tumor cells. Normal gastric epithelial cells (GES-1) show no TAG-72 expression (Figure 7B). Similarly, after incubation, the two negative control groups of the MGC80-3 cell line (Figure 7C) and the GES-1 cell line (Figure 7D) were observed to have no positive stain.

TAG-72 is a membrane protein complex that is overexpressed in a number of cancers, such as colonic adenocarcinoma, invasive ductal carcinoma of the breast, nonsmall cell lung carcinoma, epithelial ovarian carcinoma, as well as pancreatic and gastric esophageal cancers [29], with only trace levels found in histological sections of normal tissues [30,31]. In previous studies, ^131^I-labeled MAb B72.3 has demonstrated prolonged binding to human colon carcinoma xenografts, allowing *in situ* radioimmunodetection in the nude mouse model [32-35]. In this experiment, one of these second-generation MAb B72.3, CC49, was used to develop a probe to detect the TAG-72 of the gastric cancer cell line MGC80-3. Positive immunohistochemical staining (brown stain) was observed for the CC49 antibody, as expected, demonstrating that the CC49 antibody bound to MGC80-3 tumor cells, which indicated that TAG-72 is highly expressed in this tumor, while normal gastric epithelial cells (negative control) show no TAG-72 expression (Figure 7B).

### CC49-QDs Ab probe specifically binds to TAG-72 of MGC80-3 cells *in vitro*

Streptavidin peroxidase immunohistochemical analysis indicated that TAG-72 was expressed on the membrane and in the cytoplasm of MGC80-3 cells (Figure 7). Direct immunofluorescence examination with the CC49-QDs Ab probe showed that red fluorescence was present on the membrane of MGC80-3 cells in the experimental group (group B in Figure 1). In contrast, red fluorescence cannot be observed in the other groups of MGC80-3 cells (groups A and C in Figure 1) and GES-1 cell groups (groups E to G in Figure 2). These results demonstrated that the CC49-QDs Ab probe can recognize and bind efficiently to the unblocked TAG-72 of MGC80-3 cells. In contrast, MGC80-3 cells, of which TAG-72 had been blocked by the CC49 antibody (C in Figure 1) and GES-1 cells, cannot recognize and bind efficiently.

By adding QDs to CC49 antibodies, we generated a fluorescence probe directed against TAG-72 in gastric cancer cells for the first time. These alterations of the CC49 molecule did not affect the antigen-antibody reaction of CC49 and TAG-72. Also, in this experiment, the *in vitro* binding studies showed the specific binding between the CC49-QDs and the TAG-72 antigen on the MGC80-3 cells. The possibility of nonspecific binding between free QDs and MGC80-3 cells was excluded by the finding that negligible fluorescence was detected from the cells incubated with free QDs. Furthermore, excessive CC49 antibody successfully blocked the binding of CC49-QDs to the MGC80-3 cells, indicating that the binding was mediated through TAG-72. For the GES-1 cell line, neither in the CC49-QDs group nor in the free QD group could visible fluorescence be observed because of the absence of TAG-72.

The experiment has demonstrated the imaging of gastric carcinoma cells and the immunoassay of TAG-72 with near-infrared quantum dots. The optical properties and stability of these QDs and CC49-QDs have been studied. Due to the advantage of near-infrared QDs and CC49-QDs as cell imaging tools, the bioconjugation and immunofluorescent images were studied. The cell images indicate that they have a very good signal in a biotin-streptavidin labeling system. Furthermore, compared to QDs, the CC49-QDs could specifically bind to the TAG-72 of gastric cancer cells. So, the experiment demonstrates that the CC49-QDs could be another probe for gastric tumor cell detection.

## Conclusions

In conclusion, by the addition of CC49, we generated a specific QD molecule that not only has the potential to bind tumor cell *in vitro* but also could be used in a long-term therapeutic regimen to possibly alter individual cancer treatment. Further preclinical studies utilizing our CC49-QDs fusion construct, addressing the short-term and long-term capabilities, will be performed to develop regimens for improved gastric cancer treatment.

## Competing interests

The authors declare that they have no competing interests.

## Authors’ contributions

YPZ wrote the paper and finished the main work of the experiment, including QD and CC49-QDs electron microscopy and spectrum analysis, gel permeation high-performance liquid chromatography, immunohistochemical detection of TAG-72, and *in vitro* immunofluorescence. PS and WLY conceived of the idea and provided some useful suggestion. XRZ and CSS finished the former parts of the experiment such as the synthesis of CdTe QDs and CC49-QDs. All authors read and approved the final manuscript.
